# Integrated Bioinformatic Analysis of the Shared Molecular Mechanisms Between Osteoporosis and Atherosclerosis

**DOI:** 10.3389/fendo.2022.950030

**Published:** 2022-07-22

**Authors:** Liang Mo, Chao Ma, Zhangzheng Wang, Jianxiong Li, Wei He, Wei Niu, Zhengqiu Chen, Chi Zhou, Yuhao Liu

**Affiliations:** ^1^ The First Affiliated Hospital of Guangzhou University of Chinese Medicine, Guangzhou, China; ^2^ The Second Affiliated Hospital of Guangzhou University of Chinese Medicine, Guangzhou, China; ^3^ Guangdong Research Institute for Orthopedics and Traumatology of Chinese Medicine, Guangzhou, China

**Keywords:** osteoporosis, atherosclerosis, bioinformatics, molecular mechanism, inflammation, immune

## Abstract

**Background:**

Osteoporosis and atherosclerosis are common in the elderly population, conferring a heavy worldwide burden. Evidence links osteoporosis and atherosclerosis but the exact underlying common mechanism of its occurrence is unclear. The purpose of this study is to further explore the molecular mechanism between osteoporosis and atherosclerosis through integrated bioinformatic analysis.

**Methods:**

The microarray data of osteoporosis and atherosclerosis in the Gene Expression Omnibus (GEO) database were downloaded. The Weighted Gene Co-Expression Network Analysis (WGCNA) and differentially expressed genes (DEGs) analysis were used to identify the co-expression genes related to osteoporosis and atherosclerosis. In addition, the common gene targets of osteoporosis and atherosclerosis were analyzed and screened through three public databases (CTD, DISEASES, and GeneCards). Gene Ontology (GO) and Kyoto Encyclopedia of Genes and Genomes (KEGG) enrichment analyses were performed by Metascape. Then, the common microRNAs (miRNAs) in osteoporosis and atherosclerosis were screened out from the Human microRNA Disease Database (HMDD) and the target genes of whom were predicted through the miRTarbase. Finally, the common miRNAs–genes network was constructed by Cytoscape software.

**Results:**

The results of common genes analysis showed that immune and inflammatory response may be a common feature in the pathophysiology of osteoporosis and atherosclerosis. Six hub genes (namely, COL1A1, IBSP, CTSD, RAC2, MAF, and THBS1) were obtained *via* taking interaction of different analysis results. The miRNAs–genes network showed that has-let-7g might play an important role in the common mechanisms between osteoporosis and atherosclerosis.

**Conclusion:**

This study provides new sights into shared molecular mechanisms between osteoporosis and atherosclerosis. These common pathways and hub genes may offer promising clues for further experimental studies.

## Introduction

More and more pieces of evidence show that vascular system diseases are related to bone metabolism. Some scholars presented a concept of a bone-vascular axis to explore the correlation between them (1, 2). They supported that there exited cellular, endocrine, and metabolic signals that flow bidirectionally between the vasculature and bone, and vascular and skeletal disease may occur concurrently when dysmetabolic states perturbed the bone–vascular axis (2). Osteoporosis and atherosclerosis are the most common skeletal and vascular disease while also being the chronic systemic diseases and major public health problems worldwide (3). Epidemiology studies have linked osteoporosis and atherosclerosis, suggesting that postmenopausal women with osteoporosis are often accompanied by atherosclerosis (4). Decreased bone mineral density and osteoporotic fractures were significantly associated with the development of echogenic plaques in carotid artery (5). Regardless of no widely consensus on the exact cellular and molecular basis underlying the high comorbidity between osteoporosis and atherosclerosis, it has been testified that they shared risk factors, common pathogenesis and genetic factors, and a causal association (6).

Several risk factors involving ageing, postmenopausal status, smoking habit, physical inactivity, and alcohol intake were considered as the common factors shared by osteoporosis and atherosclerosis (7). Osteoblast activity and survival decline with aging, whereas osteoclast activity increases, contributing to the age-associated decline of bone mass (8). Simultaneously, atherosclerosis, a high prevalence and incidence disease in the elderly causing most heart attacks and strokes, is considered a hallmark of aging process (9). However, there is growing evidence that a potential link exists between osteoporosis and atherosclerosis beyond aging (10). Several pathophysiological mechanisms including inflammatory cytokines, lipid oxidation products, and vitamin D and K deficiency were identified for the interplay between the skeletal and vascular systems (7, 11). Inflammatory cytokines [including tumor necrosis factor–alpha (TNF-α) and interleukin (IL)-1, IL-6, and IL-17] and chemokines have been shown to be associated with atherosclerosis, increased cardiovascular morbidity and mortality, and increased bone loss (12, 13). Dyslipidemia, including elevated level of total serum cholesterol, triglycerides, and low-density lipoprotein (LDL) cholesterol, was considered to promote atherosclerosis progression and also influenced bone metabolism (14). In addition, there also exists similarity of the treatment for osteoporosis and atherosclerosis. Statins, cholesterol-lowering drugs in preventing and treating cardiovascular disease, have potential positive effects on bone mineral density and decreasing osteoporotic fracture (15). Bisphosphonate therapy for osteoporosis reduces progression of vascular calcification (16). These findings strongly suggested the interaction of these two pathological conditions, simultaneously exerting an influence on the development of osteoporosis and atherosclerosis. However, they were mainly from clinical perspectives, and few studies have investigated genomic relationship between osteoporosis and atherosclerosis.

**Figure 1:**
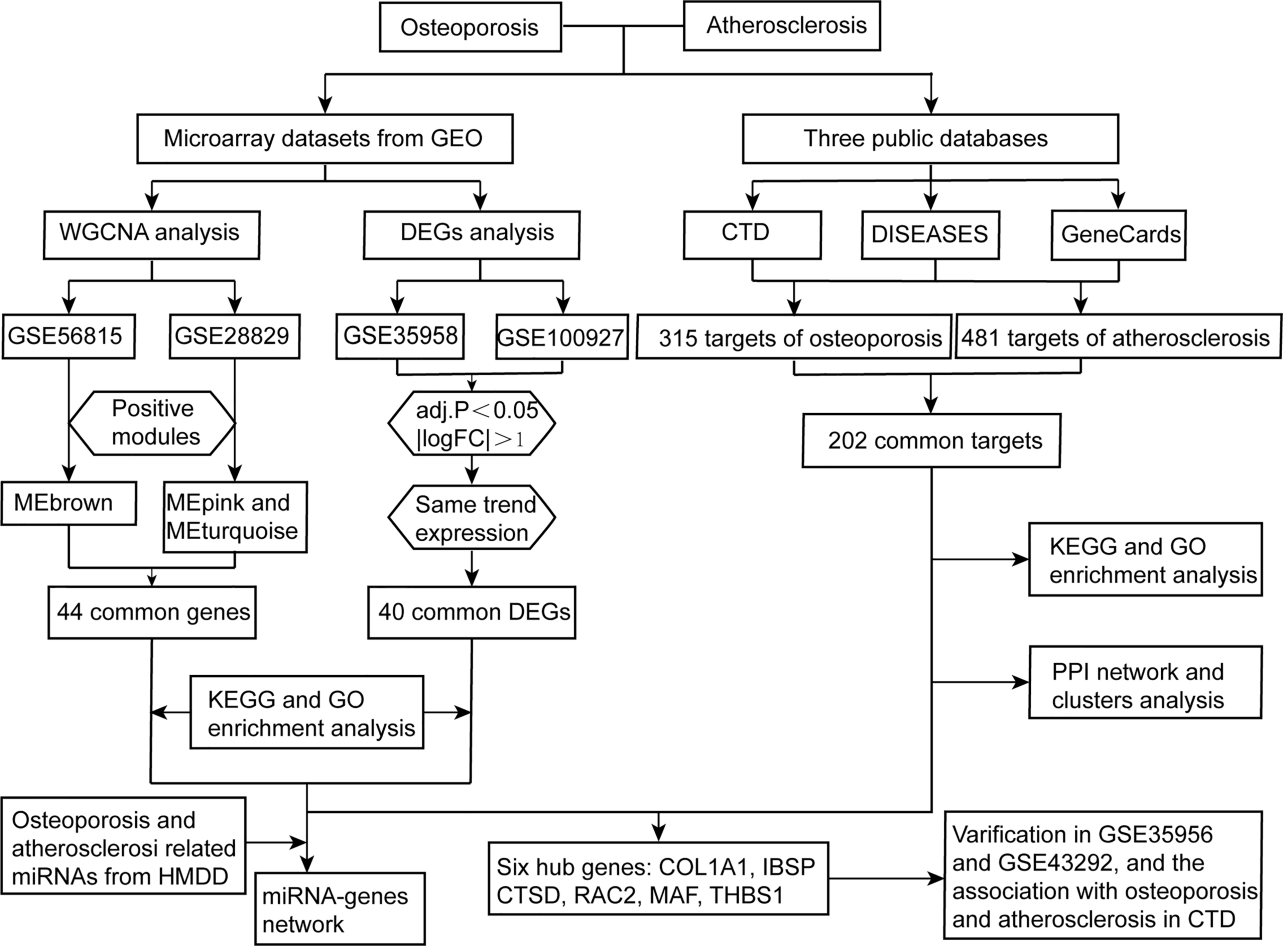
Research design flow chart.

Contemporary, the quick development of bioinformatics approaches allows us to get a better grasp of disease pathobiology more deeply from the genetic level (17). Recently, a system-level analysis of whole blood genome−wide expression data identified several enriched biological pathways and three genes (NOSIP, GXYLT2, and TRIM63) that were significantly associated with early traits of both osteoporosis and atherosclerosis, supporting the idea that they were comorbid based on transcriptomic evidence (18). However, the representativeness of their samples was limited, and studies integrating gene data from public databases with both osteoporosis and atherosclerosis are lacking. In view of this, the purpose of this study is to integrate and analyze gene data related to the pathogenesis of osteoporosis complicated with atherosclerosis from the public databases, which provide new insights into the biological mechanisms of these two diseases, and it will help to develop dual-purpose prevention methods. The research flowchart of this research was shown in **Figure 1**.

## Methods

### Data Source

Microarray datasets were downloaded from the Gene Expression Omnibus (GEO) database (http://www.ncbi.nlm.nih.gov/geo/), which contain a great deal of high-throughput sequencing and expression microarray data. The keywords “osteoporosis” and “atherosclerosis” were used to search related gene expression datasets and non-human tested specimens were excluded. Finally, the datasets numbered GSE22829, GSE56815, GSE35958, GSE100927, GSE43292, and GSE35956 were downloaded from GEO database. In addition, the co-expressed genes of osteoporosis and atherosclerosis were screened through three disease database, including Comparative Toxicogenomics Database (CTD) (http://ctdbase.org/) (19), GeneCards (https://www.genecards.org/) (20), and DISEASES (https://diseases.jensenlab.org/) database (21).

### Weighted Gene Co-Expression Network Analysis

A system biological approach called Weighted Gene Co-Expression Network Analysis (WGCNA) analysis co-expressed gene modules that have high biological significance and explores the relation between gene networks and diseases (22). Therefore, the WGCNA was used to analyze GSE56815 dataset and GSE22829 dataset to obtain the osteoporosis and atherosclerosis associated modules. Using R language, the Hclust function was used prior to the analysis of eliminating outlier samples from the hierarchical clustering analysis. Based on the criterion of R^2^ > 0.85, an appropriate soft-thresholding power β (ranged from 1 to 20) is calculated to achieve the scale-free topology. Then, through hierarchical clustering, co-expression modules were identified, and the hierarchical clustering tree was obtained. The minimum number of module genes was set as 25, and the modules were merged for the second time according to the modules with a correlation greater than 50%. Finally, the module eigengene and the correlation between the module eigengene and clinical features were calculated to obtain the expression profiles of each module. As a result, we focused on modules with high correlation coefficients with clinical features and later selected genes from these modules for further analysis.

### Analysis of Gene Modules Through WGCNA Analysis

Using the Pearson correlation coefficient and the *P*-value of eigengenes and disease traits of each module, we identified key modules in osteoporosis and atherosclerosis. Then, the genes in key modules positively associated with osteoporosis and atherosclerosis were used to obtain shared genes *via* ImageGP (23).

### Identification of Common Genes Through DEGs Analysis

GEO2R (www.ncbi.nlm.nih.gov/geo/ge2r) is an online analysis tool developed based on two R packages (GEO query and Limma) (24). The differentially expressed genes (DEGs) in GSE35958 and GSE100927 datasets were determined by comparing gene expression profiles between the diseased and control groups using GEO2R. Log|FC| > 1 and adj. *P*-value < 0.05 were considered to indicate statistical significance. The common DEGs were obtained by Venn diagram.

### Enrichment Analysis, PPI Network Construction, and Module Analysis

In order to analyze the biological functions and pathways involved in common genes, Gene Ontology (GO) and Kyoto Encyclopedia of Genes and Genomes (KEGG) enrichment analyses were performed using Metascape, which is a web-based portal designed to provide a comprehensive gene list annotation and analysis resource for experimental biologists (25). Min overlap = 3 and Min Enrichment = 1.5 were the screening conditions. The *P-*value < 0.01 was considered significant.

The protein–protein interaction (PPI) network was analyzed using the Search Tool for the Retrieval of Interacting Genes (STRING; http://string-db.org). An interaction with a combined score > 0.4 was selected and used to construct a PPI network with Cytoscape software (version 3.7.0). The cluster analysis used Cytoscape’s plug-in molecular complex detection technology (MCODE) with default parameters: K-core = 2, degree cutoff = 2, max depth = 100, and node score cutoff = 0.2.

### Shared Gene Targets Obtained From Public Database

The common osteoporosis-related genes and the atherosclerosis-related genes that were shared between the three public databases were obtained using the Venn diagram. Then, taking the intersection of them, we obtained the common gene targets between osteoporosis and atherosclerosis.

### Hub Gene Selection and Validation

Common gene targets between osteoporosis and atherosclerosis were obtained through disease database screening. At the same time, shared genes were also gained from WGCNA and DEG analysis. Then, the intersection of common targets from disease databases and genes from WGCNA and DEG analysis was selected as hub genes. Furthermore, the expression of hub genes was verified in GSE43292 and GSE35956. The comparison between the two sets of data was performed with the t-test. *P*-value < 0.05 was considered significant.

### Hub Gene Interaction With Diseases

In order to explore the relationship between hub genes and diseases, CTD was used to isolate the inference score and reference count of hub genes associated with osteoporosis and atherosclerosis. Interaction between hub genes and atherosclerosis, cardiovascular diseases, vascular diseases, osteoporosis, bone diseases, and bone resorption was analyzed in CTD. The inference score and reference count were visualized by the Histogram.

### Identified the Common miRNAs

Small non-coding RNAs called microRNAs can modulate gene expression by promoting or inhibiting mRNA degradation and translation (26). We therefore investigate whether some miRNAs share a common regulatory mechanism and development process in osteoporosis and atherosclerosis. Osteoporosis-associated and atherosclerosis-associated miRNAs were obtained from the Human microRNA Disease Database (HMDD), which presents more detailed and comprehensive annotations to the human miRNA-disease association data, including miRNA-disease association data from the evidence of genetics, epigenetics, circulating miRNAs, and miRNA-target interactions (27). Based on a published literature, we further identified the expression levels of these miRNAs in osteoporosis and atherosclerosis, and only miRNAs with the same disorder types were further analyzed. In particular, GO analysis of these common miRNAs was performed using the online software mirPath (v.3) from DIANA tools. The GO terms with *P*-values < 0.01 were considered significant.

### The Common miRNAs–Genes Network Construction

Target gene information of common miRNAs was collected from miRTarbase (http://mirtarbase.mbc.nctu.edu.tw/php/index.php), which is an experimentally validated miRNA-target interactions database (28). The intersection of target genes of common miRNAs and shared genes in osteoporosis and atherosclerosis was used to construct the miRNAs–genes regulated network. Cytoscape software was used to visualize the network.

## Results

### GEO Information

Six GEO datasets (namely, GSE56815, GSE28829, GSE100927, GSE35958, GSE43292, and GSE35956) were selected in all. Detailed information of these six datasets is shown in **Table 1**, such as GSE number, detection platforms, and samples. GSE56815 and GSE28829 were paired used for the WGCNA analysis, GSE35958 and GSE100927 were paired used for the DEG analysis, and GSE35956 and GSE43292 were used to verify the hub gene expression levels.

**Table 1:** Detailed information of GEO datasets.

ID	GSE number	Platform	Samples	Disease
1	GSE56815	GPL96	40 patients and 40 controls	osteoporosis
2	GSE35958	GPL570	4 patients and 5 controls	osteoporosis
3	GSE35956	GPL570	5 patients and 5 controls	osteoporosis
4	GSE28829	GPL199	16 patients and 13 controls	atherosclerosis
5	GSE100927	GPL17077	69 patients and 35 controls	atherosclerosis
6	GSE43292	GPL6244	32 patients and 32 controls	atherosclerosis

### The Co-Expression Modules in Two Diseases

**Figure 2:**
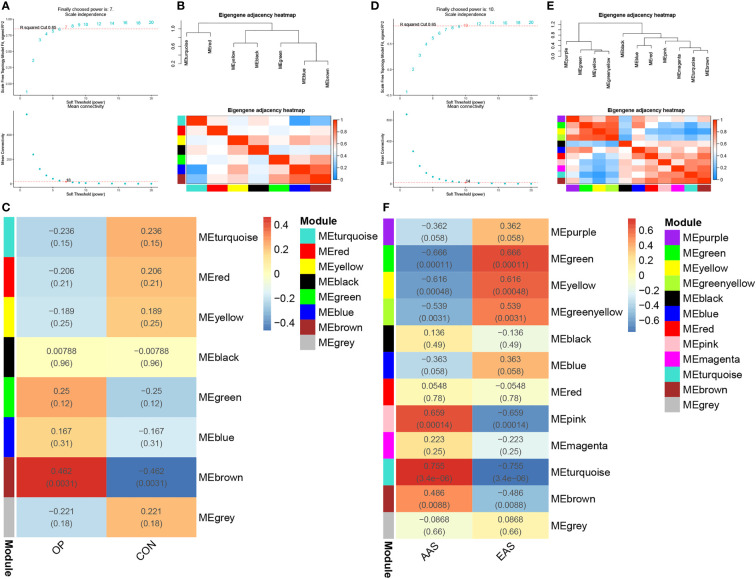
Weighted genes correlation network analysis (WGCNA) of GSE56815 and GSE28829 datasets. **(A)** Soft threshold analysis in osteoporosis. **(B)** Module correlations in osteoporosis. **(C)** Heatmap of the module–trait relationship in osteoporosis. Each cell contains the corresponding correlation and *P*-value. **(D)** Soft threshold analysis in atherosclerosis. **(E)** Module correlations in atherosclerosis. **(F)** Heatmap of the module–trait relationship in atherosclerosis. Each cell contains the corresponding correlation and *P*-value. OP, osteoporosis; CON, control; AAS, advanced atherosclerosis; EAS, early atherosclerosis.

As shown in **Figures 2A–C**, gene modules associated with the bone mineral density phenotype were obtained by WGCNA analysis of GSE56815 dataset, including circulating monocytes from females with 40 high bone mineral density and 40 low bone mineral density. Clustering analysis of GSE56815 showed that the GSM1369791 sample was poorly clustered (**Supplementary Figure 1A, B**). Therefore, this sample was excluded as an outlier in the WGCNA analysis. The analysis of soft threshold selection revealed that gene associations were maximally consistent with the scale-free distribution and when β = 7 (scale free R^2^ = 0:85). Then, a total of eight modules were identified in the weighted gene co-expression network by merging modules with feature factors greater than 0.5 and setting the minimum number of genes in a module to 25. We found that, as shown in **Figure 2C**, MEbrown module (r = 0.462, p = 0.0031) was positively correlated with osteoporosis in non-gray modules. Genes in the MEbrown module were further used to the analysis, including 257 genes.

As shown in **Figures 2D–F**, a total of 12 gene modules were obtained through the WGCNA analysis of GSE28829 dataset, including 16 advanced atherosclerotic segments and 13 early atherosclerotic segments from human. Clustering analysis showed that no sample was excluded as an outlier in the WGCNA analysis (**Supplementary Figures 1C, D**). Similarly, a heatmap was mapped about module–trait relationships according to the Spearman’s correlation coefficient to evaluate the association between each module and the disease. Among these 12 modules, the correlations of two modules “MEturquoise” and “MEpink” was high and they were positively correlated with atherosclerosis (MEturquoise module: r = 0.755, p = 3.4e-06; MEpink modules: r = 0.659, p = 0.00014), including 446 and 69 genes, respectively (**Figure 2F**).

### Enrichment Analysis of Common Genes from WGCNA

**Figure 3:**
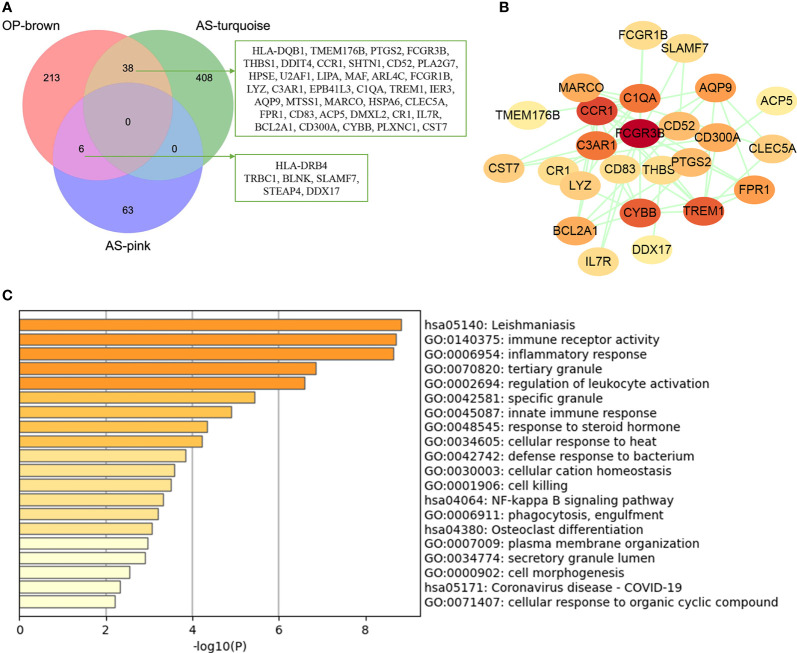
Analysis of shared genes through WGCNA. **(A)** The shared genes between the MEpink and MEturquoise modules of atherosclerosis and MEbrown module of osteoporosis by overlapping them. **(B)** The PPI network of the shared genes. **(C)** GO and KEGG enrichment analysis of the shared genes. OP, osteoporosis; AS, atherosclerosis.

The common genes were screened between atherosclerosis positively related modules (MEturquoise and MEpink modules) and osteoporosis positively related modules (MEbrown module). Then, 44 common genes were identified in the three positivity related modules of osteoporosis and atherosclerosis (**Figure 3A**). PPI network was further constructed by Cytoscape (**Figure 3B**). To explore the potential functions of these genes, GO and KEGG enrichment analyses were performed using Metascape. Results showed that these common genes were mainly enriched in leishmaniasis, immune receptor activity, inflammatory response, tertiary granule, and regulation of leukocyte activation, which indicated that most of them were involved in immune- and inflammatory-related functions (**Figure 3C**).

### The Unique Gene Signatures in Two Diseases

**Figure 4:**
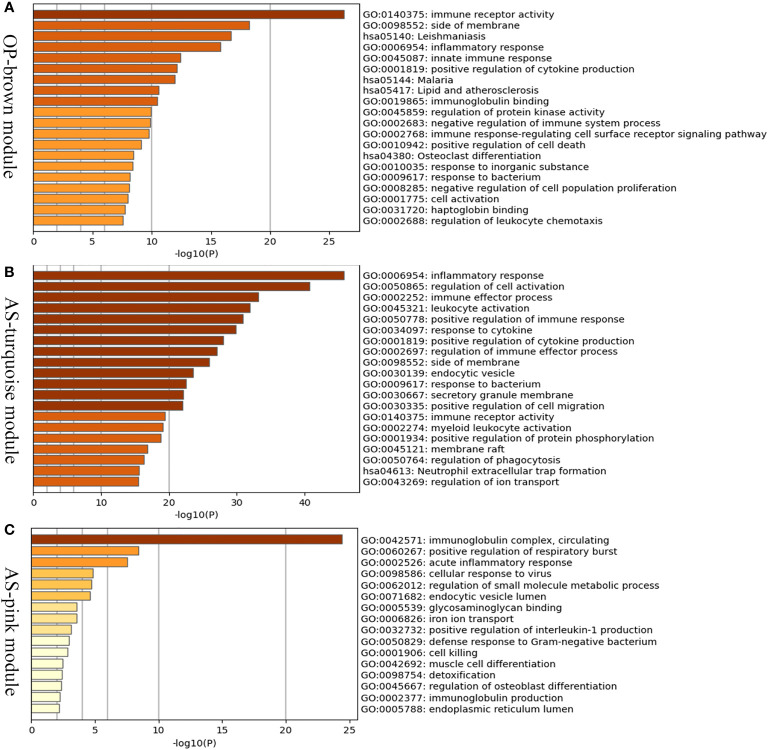
Enrichment analysis of positive related modules in osteoporosis and atherosclerosis. **(A)** GO and KEGG enrichment analysis of MEbrown module in osteoporosis. **(B)** GO and KEGG enrichment analysis of MEturquoise module in atherosclerosis. **(C)** GO and KEGG enrichment analysis of MEpink module in atherosclerosis. OP, osteoporosis; AS, atherosclerosis.

In order to investigate the possible pathogenesis of osteoporosis and atherosclerosis, enrichment analysis of genes in their positive modules was performed. Function enrichment analysis showed that the genes in osteoporosis MEbrown module were mainly associated with immune and inflammatory response. Interestingly, we also found that they were also mainly enriched in lipid and atherosclerosis pathway, which is closely associated with the development and progression of atherosclerosis. Therefore, it may hint that a close link exists between osteoporosis and atherosclerosis at the molecular level (**Figure 4A**).

The MEturquoise and MEpink modules were closely associated with atherosclerosis, and the genes shared with osteoporosis were mainly located in the MEturquoise module. As shown in the **Figures 4B, C**, the turquoise and pink modules were also mainly related to inflammatory response, immune effector process, and leukocyte activation. The results described above indicated that immune and inflammatory response may play important roles in both osteoporosis and atherosclerosis and be major contributors to atherosclerosis complicted with osteoporosis.

### Identification and Analysis of Common DEGs

**Figure 5:**
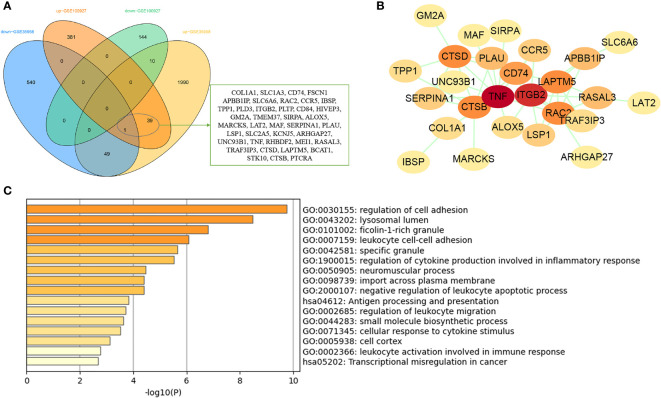
Analysis of common DEGs between osteoporosis and atherosclerosis. **(A)** The Venn diagram of the common DEGs in GSE35958 and GSE100927. **(B)** The PPI network of the common DEGs. **(C)** GO and KEGG enrichment analysis of the common DEGs.

GSE35958 and GSE100927 datasets were used to DEG analysis. The GSE35958 dataset contains four osteoporosis mesenchymal stem cell samples and five non-osteoporosis mesenchymal stem cell samples from human bone marrow. GSE100927 consists of 69 atherosclerotic plaque samples and 35 control artery samples from human peripheral arteries. After analysis of GEO2R, a total of 575 and 2,619 DEGs were identified in GSE100927 and GSE35958, respectively. The overall distribution of DEGs (fold change > 1 and adj. *P-*value < 0.05) was reflected by the volcanic map (**Supplementary Figure 2**). After taking the intersection of the Venn diagram, 40 common upregulated DEGs in both datasets were obtained (**Figure 5A**). PPI network showed that TNF, ITGB2, CTSD, and LAPTM5 had high degree (**Figure 5B**). Function enrichment analysis indicated that these common DEGs were enriched in regulation of cell adhesion, lysosomal lumen, specific granule, and inflammatory response (**Figure 5C**). Interestingly, we found that immune and inflammatory response–related functions were significantly enriched again, which were consistent with the results of WGCNA analysis.

### Analysis of Common Gene Targets From Three Public Databases

**Figure 6:**
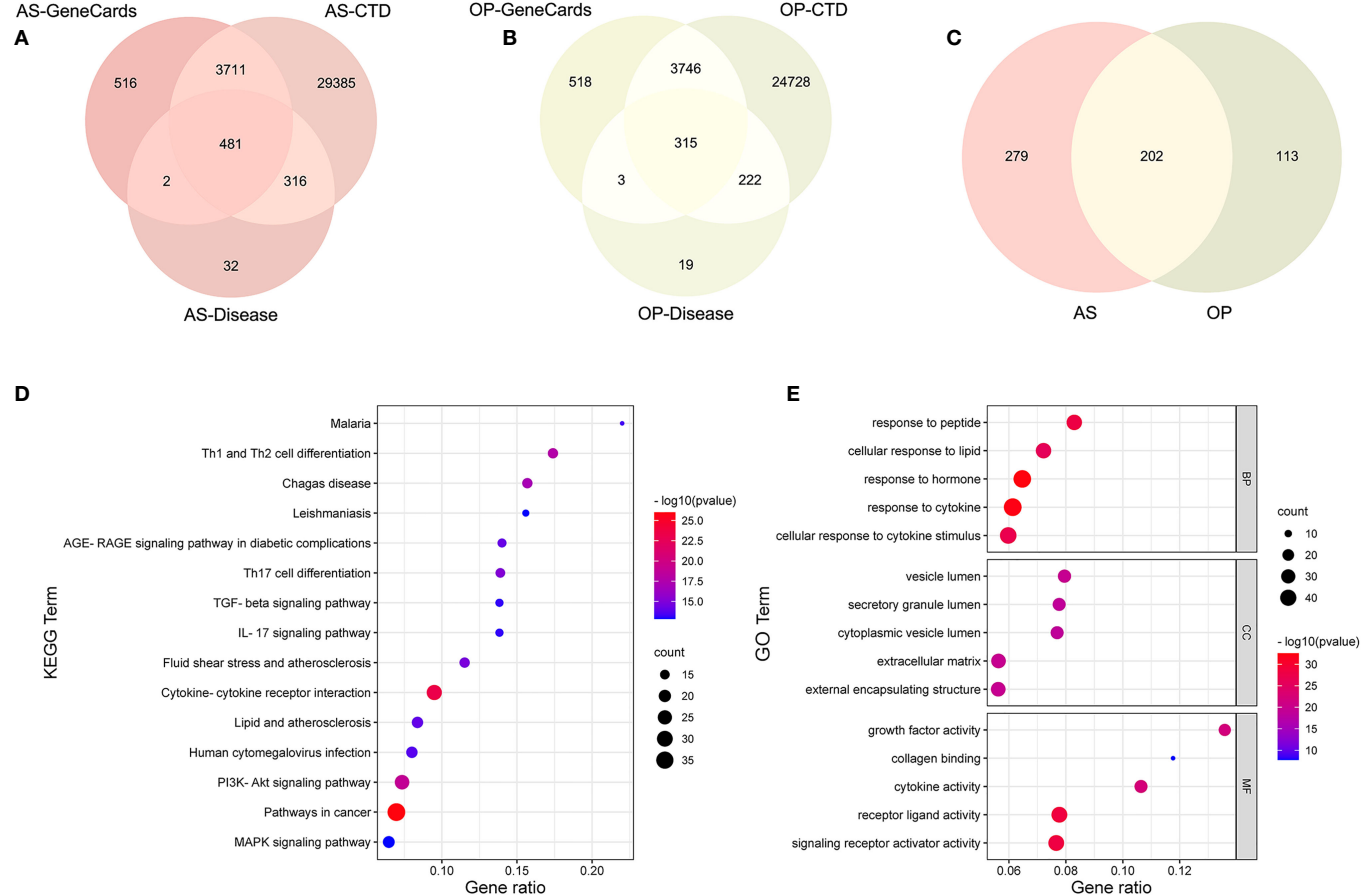
Analysis of common targets between osteoporosis and atherosclerosis from three public databases. **(A–C)** Venn diagram of common targets between atherosclerosis and osteoporosis. **(D)** KEGG enrichment analysis of the common targets. **(E)** GO enrichment analysis of the common targets.

In order to integrate the reported biological data, we combined osteoporosis- and atherosclerosis-related genes available in CTD, DISEASES, and GeneCards databases by Venn diagram software, respectively. Then, 315 genes related to osteoporosis and 481 genes to atherosclerosis were selected, and 202 common gene targets between osteoporosis and atherosclerosis were mapped, which hints that osteoporosis and atherosclerosis share a large common set of genes (**Figures 6A–C**). The list of 202 common gene targets of osteoporosis and atherosclerosis was showed in **Supplementary Table 1**.

KEGG enrichment analysis of these 202 common genes revealed that most of the shared genes related pathways are linked to lipid and atherosclerosis, PI3K-Akt signaling pathway, and some immune and inflammation-related pathways, including cytokine–cytokine receptor interaction, IL-17 signaling pathway, and T-cell differentiation, which were consistent with our enrichment analysis results above. **Figure 6D** contains the top 15 results and **Supplementary Table 2** contains the top 20 signalling pathways. **Figure 6E** contains the top five results of three GO enrichment analysis, including biological process (BP), cellular component (CC), and molecular function (MF). **Supplementary Table 3** contains the top 20 results of BP analysis results.

**Figure 7:**
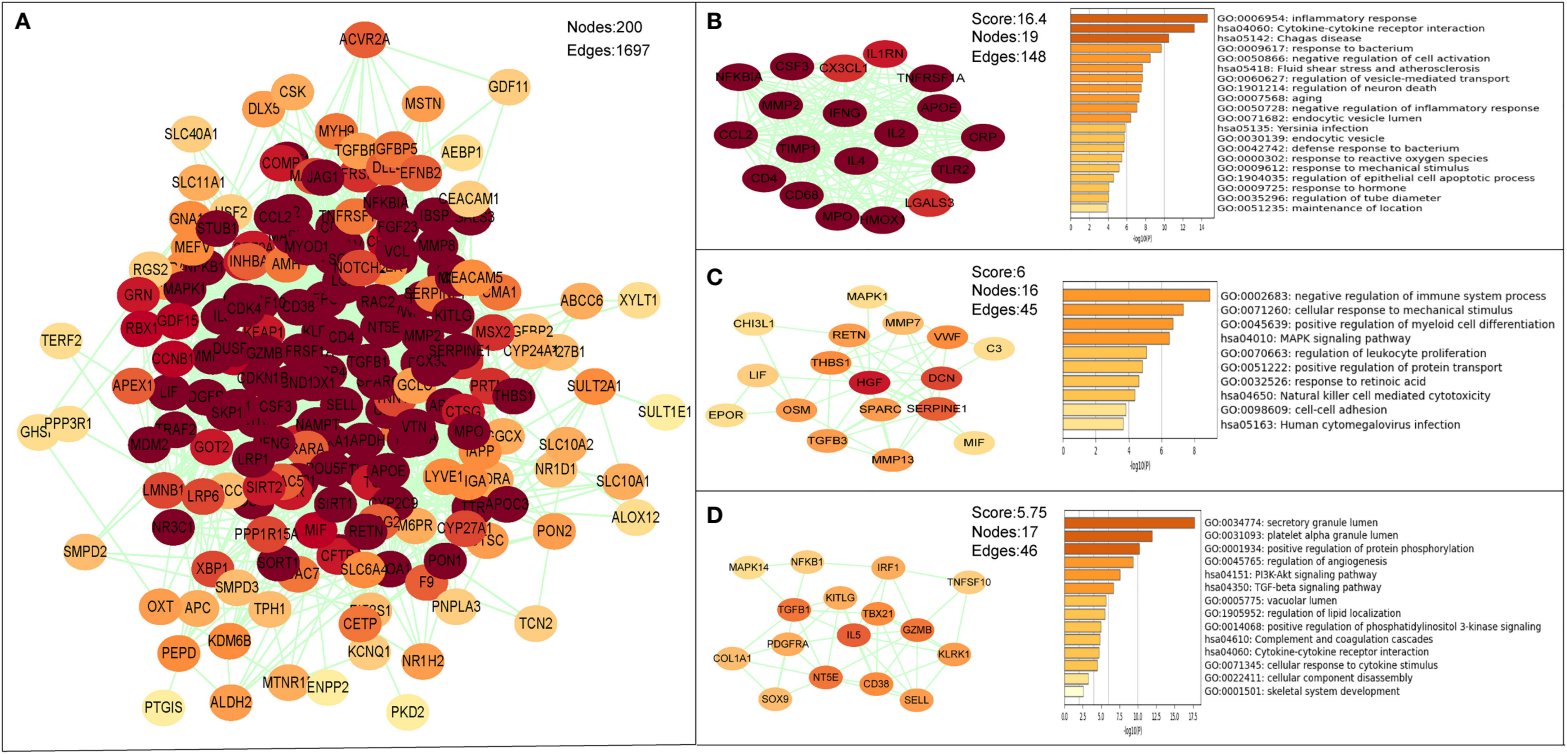
The PPI network and clusters analysis of common targets. **(A)** PPI network of 202 common targets. **(B–D)** Three significant gene clustering modules and enrichment analysis of the modular genes.

The PPI network of the common targets was constructed with combined scores greater than 0.4 using Cytoscape, which contained 200 nodes and 1,697 edges (**Figure 7A**). Three closely connected gene modules were obtained through MCODE plug-in of Cytoscape (**Figures 7B–D**). Enrichment analysis results of these three clusters showed that the immune and inflammatory response–related functions were enriched again.

### Identification and Analysis of Hub Genes

**Figure 8:**
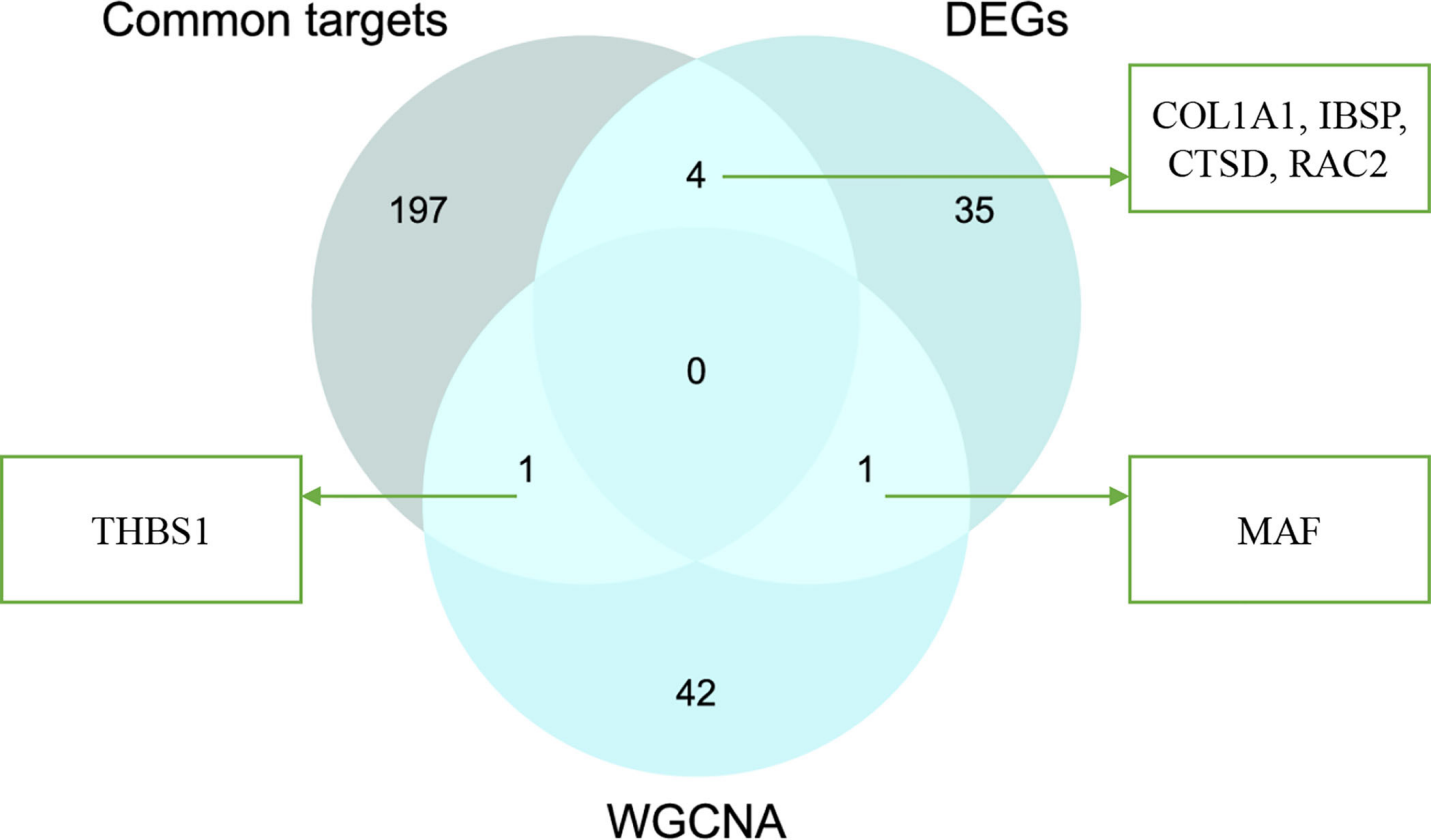
Venn diagram of interaction between common targets and genes from WGCNA and DEG analysis.

**Figure 9:**
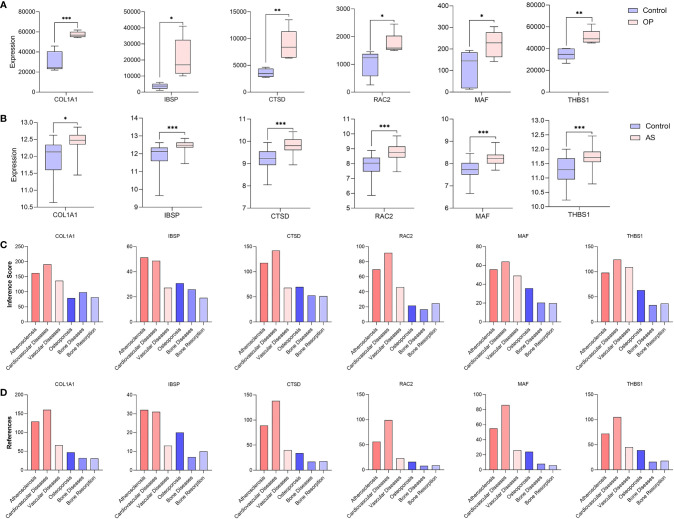
Verification of hub genes. **(A)** The expression level of hub genes in GSE35956. **(B)** The expression level of hub genes in GSE43292. The comparison between the two sets of data uses the mean t-test; *P*-value < 0.05 was considered statistically significant. *p < 0.05; **p < 0.01; ***p < 0.001. Inference score **(C)** and reference count **(D)** between hub genes and atherosclerosis, cardiovascular diseases, vascular diseases, osteoporosis, bone diseases, and bone resorption in CTD.

After taking the intersection of three sets, six hub genes (COL1A1, IBSP, CTSD, RAC2, MAF, and THBS1) were identified (**Figure 8**). In order to verify the reliability of these hub gene expression levels, we then selected GSE35956 dataset to analyze the expression levels of these hub genes in osteoporosis, and GSE43292 dataset to analyze the expression levels of hub genes in atherosclerosis. Interestingly, all hub genes were significantly upregulated in both the osteoporosis and atherosclerosis group compared with the control group (**Figures 9A, B**).

In addition, we find that these six hub genes were associated with not only osteoporosis but also atherosclerosis with different inference scores and reference counts in CTD (**Table 2**). The information of interaction between hub genes and diseases was shown in **Figures 9C, D**.

**Table 2:** The hub genes associated with atherosclerosis and osteoporosis in CTD.

Gene/Disease	Atherosclerosis		Osteoporosis	
	Inference Score	References	Inference Score	References
COL1A1	162	129	79.32	47
RAC2	69.82	56	21.68	16
THBS1	98.13	72	63.13	39
MAF	55.78	55	35.76	24
CTSD	117.68	89	69.85	34
IBSP	51.38	32	30.75	20

### Identification and Analysis of Common miRNAs in Two Diseases

A total of 119 miRNAs associated with atherosclerosis and 32 miRNAs associated with osteoporosis were screened out from the HMDD database (**Supplementary Data 1**). After taking the intersection of them, 17 common miRNAs between osteoporosis and atherosclerosis were obtained. According to the published literature provided by the HMDD database, we obtained the disorder types of these common miRNAs; there were six miRNAs (hsa-miR-133b, hsa-miR-205-5p, hsa-miR-21-3p, hsa-miR-320a, hsa-miR-23b-3p, and hsa-miR-181a-5p) upregulated and two miRNAs (hsa-miR-150-5p and hsa-let-7g-5p) downregulated in both osteoporosis and atherosclerosis. Then, the eight miRNAs were further studied. The enrichment analysis showed that the functions of these miRNAs were involved in multiple BPs including immune response related functions (**Supplementary Figure 3**).

### The common miRNAs-shared genes network

**Figure 10:**
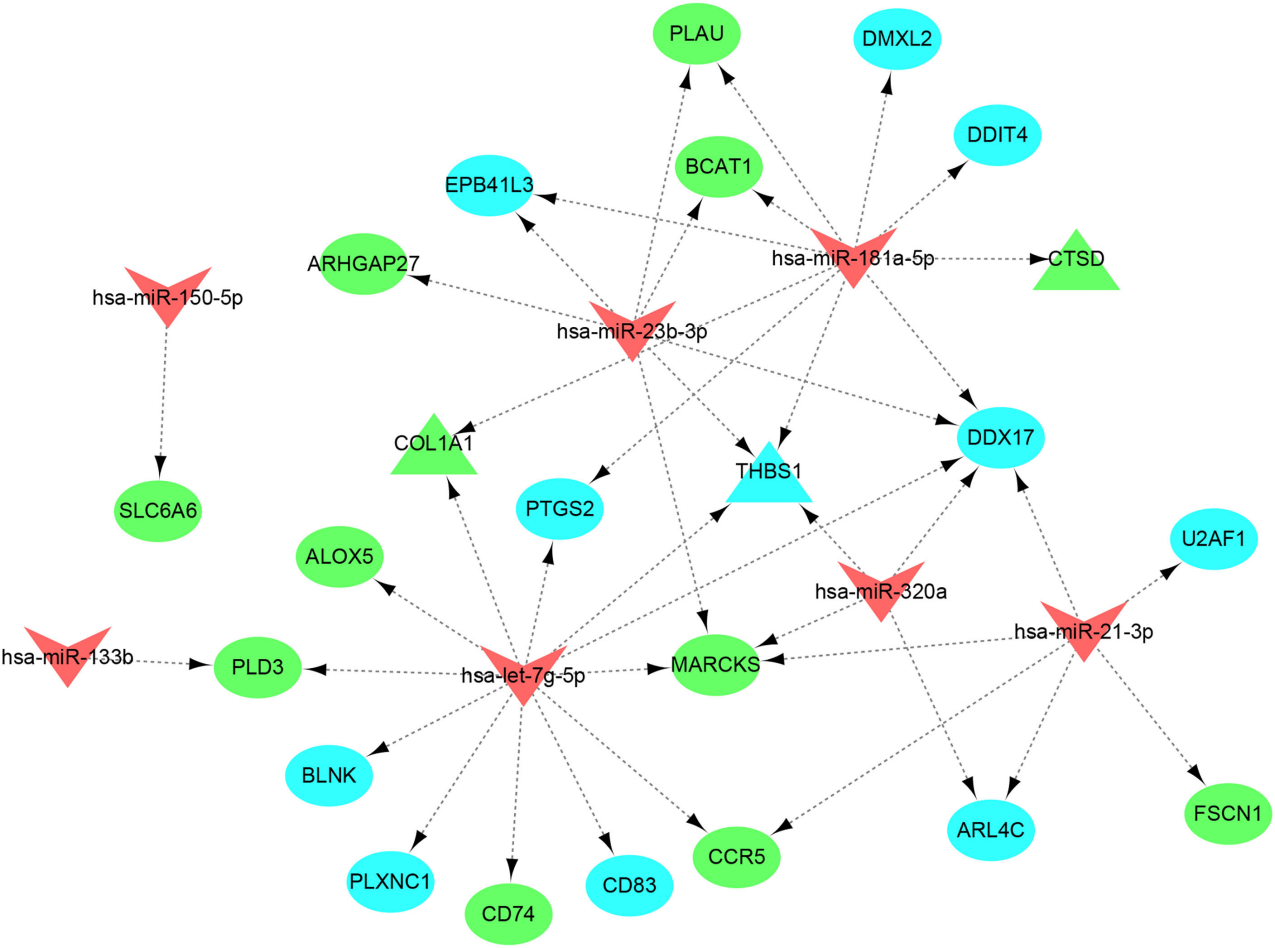
miRNAs–shared genes regulatory network. The V-shape represents miRNA, ellipse shape represents gene, and triangle shape represents hub gene. Blue represents gene from WGCNA and green represent gene from DEGs.

A total of 5,015 target genes of eight common miRNAs were predicted using miRTarbase, and the miRNAs–genes network was constructed by taking the intersection of them and shared genes (obtained from WGCNA and DEGs). Finally, the miRNAs–genes network contained seven miRNAs and 23 shared genes, including three hub genes (CTSD, COL1A1, and THBS1) (**Figure 10**). It is evident from the network that hsa-let-7g-5p regulated the most downstream target genes. Moreover, target genes predicted by has-let-7g were closely related to the inflammatory and immune response using the online software mirPath (v.3) from DIANA tools. Therefore, we speculate that has-let-7g might function importantly in the common mechanism of osteoporosis and atherosclerosis.

## Discussion

Osteoporosis and atherosclerosis are both widely prevalent disorders and often seen concurrently, exerting a severe impact on public health (29). The common pathophysiological mechanisms between osteoporosis and atherosclerosis have been attracting intense research interest around the world. Previous studies identified some molecular lipid species associated with early markers of both osteoporosis and atherosclerosis based on the Young Finns Study cohort, supporting the osteoporosis and atherosclerosis comorbidity hypothesis (14, 30). Latest research found that platelet-derived growth factor–BB (PDGF-BB), secreted from preosteoclasts, worked as an important mediator of vascular stiffening in response to aging and metabolic stress (31). Extracellular vesicles derived from aged bone matrix during bone resorption promote bone marrow mesenchymal stem cells adipogenesis rather than osteogenesis and augment calcification of vascular smooth muscle cells (32). These results have deepened our knowledge of the concept of a bone-vascular axis and may help to reveal the molecular mechanism between osteoporosis and atherosclerosis. However, it seems that few studies have explore the common pathogenesis of osteoporosis and atherosclerosis on a genetic level. To our knowledge, this is the first time to investigate osteoporosis and atherosclerosis comorbidity hypothesis by integrating data from a variety of public databases to identify the common mechanisms of osteoporosis and atherosclerosis.

Global gene expression studies can help us better understand the specific pathobiology between osteoporosis and atherosclerosis. The results of WGCNA and DEG analysis showed that immune and inflammatory response–related functions may play an important role in both osteoporosis and atherosclerosis. At the same time, we found that T-cell differentiation and inflammatory signaling pathways were also enriched in the enrichment analysis results of common genes from public disease databases. Therefore, an inflammatory environment caused by the immune and inflammatory response may be a common feature in the pathophysiology of osteoporosis and atherosclerosis.

Under physiological conditions, the bone homeostasis is regulated by osteoblast-mediated bone formation and osteoclast-mediated bone resorption through receptor activator of nuclear factor–κB (RANK), ligand for a RANK receptor (RANKL), and osteoprotegerin (OPG) interactions (33). Any imbalance of this control leads to an increase in the activity of osteoclast, resulting in osteoporosis. Although initially, it was thought that hormonal imbalance was the leading cause of osteoporosis, the role of the immune system in osteoporosis slowly came into view with advanced studies (34, 35). In pathological states such as rheumatoid arthritis, multiple sclerosis, osteoarthritis, and bone tumors, a host of inflammatory cytokines and activated immune cells disrupts this balance in favor of osteoclast-mediated bone resorption (36). Accumulated pieces of evidence suggested that immune and inflammatory response induced by both innate and adaptive immune cells can affect bone metabolism through several pathways.

Immune cell activation often goes along with inflammatory mediators’ production, such as reactive oxygen species, and pro-inflammatory cytokines and chemokines, which directly or indirectly influence bone metabolism and promote the development of osteoporosis (37). In innate immune system, pro-inflammatory cytokines such as TNF-a and IL-6 can stimulate the polarization of macrophages into M1 macrophages (both an inflammatory phenotype and a precursor of osteoclast), associating with bone catabolic activity (38). The monocytes show high levels of C-C chemokine receptor 2 (CCR2) in inflammatory microenvironment and can serve as osteoclast precursor, participating in bone remodeling by producing cytokines (39). In addition, dendritic cells and neutrophils can activate T cells, and the activated T cells produce cytokines and soluble factors that participate in bone resorption process (40). In adaptive immune system, T cells are major players of it and the results of our enrichment analysis were also involved in T_H_1, T_H_2, and T_H_17 cell differentiation and inflammatory factor–related pathways. Previous studies have found that T_H_1 and T_H_2 cells inhibit osteoclastogenesis *via* secreting IFN-γ and IL-4 cytokines and thus act as an osteoprotective role (41). Lower serum levels of IFN-γ and IL-4 cytokines in postmenopausal osteoporotic patients further suggest its osteoprotective role (42). However, a study suggested that IFN-γ can also promote osteoblasts generation by inducing expression of RANKL on activated T cells and thus plays a dual role in bone remodeling (43). T_H_17 cells are osteoclastogenic subsets of T cells and can produce proinflammatory cytokine, including RANKL, TNF-α, IL-17, and IL-6, all of which augment osteoclastogenesis (44). Moreover, enhancement in the number of T_H_17 cells and enhanced expression of proinflammatory cytokines (IL-6, TNF-α, RANKL, and IL-17) were observed in osteoporotic mice (45). Recent research suggested that “pyroptosis” of osteoblast (a programmed cell death mechanism) correlates with inflammation and contributes to excessive differentiation of osteoclasts *via* producing NLRP3 and also IL-1β and IL-18 (46). In summary, the concept of osteoimmunology was consistent with the enrichment results above in our study. Zhang et al. (47) performed a GO analysis of genes corresponding to differentially expressed proteins in osteoporosis and also found that immune inflammation and related functions were significantly enriched, consistent with our analysis.

Atherosclerosis is a chronic disease with an autoimmune component due to its accompaniment with a chronic, low-grade inflammatory response that attracts cells of the innate and adaptive immune systems into the atherosclerotic plaque (48). The important role of immunity and inflammation in atherosclerosis has been demonstrated by overwhelming experimental and clinical evidence. In our study, the analysis of positively correlated modules in atherosclerosis also supported this view. A genome-wide association study for coronary artery disease identified some locus linked to inflammation (49). The most robust genetic association has been identified for single-nucleotide polymorphisms in the 9p21 locus, which has been implicated in regulation of IFN-γ signaling (50). Oxidized LDL (oxLDL), the well-established precursor particles of atherosclerosis, can trigger inflammation of the arterial wall by binding to Toll-like receptors (TLRs) (51), and it can also reduce osteoblast viability (52).

In the vessel wall, oxLDL and inflammatory statement elicit an influx of monocytes that differentiate into macrophages and then into foam cells, accumulate intracellular cholesterol, and produce inflammatory mediators (53). CD4^+^ T cells are also recruited to the forming lesion, and single-cell data from human atherosclerotic plaques showed that the majority of CD4^+^ T cells in the plaque are T_H_1 and T_H_2 cells (54). In our study, we similarly found a significant enrichment for T_H_1 and T_H_2 cell differentiation pathway. Proinflammatory mediators such as IFN-γ, TNF-α, IL-2, and IL-3, produced by T_H_1 and T_H_2 cells, can activate macrophages and other plaque cells and thereby accelerate the inflammatory response (54). Knocking IFN-γ and its receptor protects mice from atherosclerosis (55). In addition, T_H_17 cells also can be induced by oxLDL and constitute a minor population in plaques, promoting the vascular inflammation (56). Ongoing inflammatory and hemodynamic assaults on the atherosclerotic lesion might eventually cause local dysfunction or breakdown of endothelial integrity. One study identified the DEGs in atherosclerosis, compared with healthy controls. The bioinformatics analysis showed that immune response, inflammatory response, and vascular smooth muscle contraction were the unique gene signatures in atherosclerosis, which were consistent with our analysis (57).

As mentioned above, we found that immune and inflammatory response were enrich in both atherosclerosis and osteoporosis, and most of common genes obtained from WGCNA, DEGs, and three public databases were associated with immune response. The immune-associated genes in both atherosclerosis and osteoporosis from WGCNA mainly included TREM1, CYBB, CCR1, CD83, CD52, IL7R, and THBS1. The immune-associated genes from DEGs mainly included TNF, ITGB2, CD74, CCR5, and MAF. The immune-associated genes from three public databases mainly included IFNG, IL2, IL4, CCL2, TGFB1, and TLR2. Therefore, the occurrence and development of atherosclerosis and osteoporosis are complicated and these genes may provide clues for the common underlying mechanisms between them. Suppression of inflammation may have beneficial effects on bone and on vasculature.

Then, six hub genes (COL1A1, IBSP, CTSD, RAC2, MAF, and THBS1) were obtained through the integration of multisource databases and they were all significantly upregulated in both the osteoporosis and atherosclerosis group compared with the control group. According to CTD, COL1A1 was closely associated with osteoporosis and atherosclerosis and it had the highest inference score and reference count. COL1A1 is mainly involved in bone matrix formation, coding for collagen type 1 which is the most abundant extracellular protein in bone and serves as an indicator of bone formation (58). Mutations in COL1A1 gene have been demonstrated to be responsible for the autosomal dominant form of osteogenesis imperfecta, with severe osteoporosis (59). Studies have confirmed that COL1A1 is overexpressed in artery at animals with atherogenic diet and the increase was contributed to the inflammatory process and the activate the cytokine TGF-β, which supports the fibrotic process through expressing COL1A1 (60). In addition, previous studies have shown that COL1A1 may influence the prognosis in tumors by affecting infiltrating immune cells (61). Therefore, COL1A1 may be involved in the pathogenesis of osteoporosis and atherosclerosis by influencing bone formation, vascular fibrosis, immune response, but these related molecular mechanisms warrant additional investigation. Thrombospondin-1 (THBS1), an immune-associated gene, can affect endothelial cell proliferation, migration, and apoptosis by antagonizing the activity of VEGF, participating in the regulation of vascular formation (62). The latest research suggests that CD68^+^ macrophages activate TGF-β1 by expression and secretion of THBS1 and consequently induce bone information (63). Importantly, THBS1 was closely related to inflammatory response and is found to be elevated in inflammatory processes (64). However, few studies directly analyze the role of THBS1 in osteoporosis and atherosclerosis, which emphasizes its importance in future research. Other hub genes were also involved in the pathogenesis of osteoporosis and atherosclerosis by regulating different BPs and believe that the specific mechanism is worthy of further exploration.

Finally, by constructing miRNAs–genes network, we found that let-7g had the most target genes and it also involved in immune and inflammatory response. It was reported that the let-7g was significantly downregulated in patients with recent osteoporotic fractures and it could enhance osteoblast formation *in vitro* and *in vivo* by targeting HMGA2 (65). In addition, it also can regulate osteoblast formation by targeting COL1A2 (66). More importantly, high-fat diet can suppress let-7g expression (67) and activate NF-κB signaling pathway, which serves as a pivotal mediator of inflammatory responses (68). let-7g can reduce macrophage transformation and alleviates foam cell apoptosis by suppressing NF-κB pathways to prevent atherosclerosis (69). Because the expression of let-7g is regulated by a number of cellular elements which are associated with inflammatory states and chemokine release (70), it is possible that inflammatory conditions contribute to the relative paucity of let-7g resulting in reducing osteogenic formation and increasing endothelial and foam cell apoptosis, in favor of both osteoporosis and atherosclerosis. In view of the critical role of let-7g in inflammatory response, let-7g might be an important potential target for the treatment of osteoporosis and atherosclerosis.

In conclusion, our work revealed that the immune and inflammatory response might be a common susceptible factor for both osteoporosis and atherosclerosis and identified novel gene candidates who could be used as biomarkers or as potential therapeutic targets. It may provide some clues for detailed molecular mechanisms underlying bone–vascular axis. However, the results of our study need to be further verified in cell or animal experiments, which will be critical direction for future research.

## Data Availability Statement

The original contributions presented in the study are included in the article/**Supplementary Material**. Further inquiries can be directed to the corresponding authors.

## Author Contributions

LM, CM, and YL contributed to the conception of the study; LM, CM, ZW, and JL performed the analysis; CZ, WN, ZC, and WH contributed significantly to analysis and manuscript preparation; LM and CM performed the data analyses and wrote the manuscript; LM, WH, ZC, CZ and YL helped perform the analysis and constructive discussions. YL, CZ, and ZC gained the founding; All authors contributed to the article and approved the submitted version.

## Funding

This research was supported by Guangzhou Science and Technology Bureau (Grant No. 202102020930), National Natural Science Foundation of China (Grant No. 82104883, No. 81573996), Natural Science Foundation of Guangdong Province (Grant No. 2021A1515011484), Traditional Chinese Medicine Bureau of Guangdong Province (Grant No. 20221136) and Double First-rate Discipline and High-level University Construction Projects of Guangzhou University of Chinese Medicine (Grant No. 2021xk46).

## Conflict of Interest

The authors declare that the research was conducted in the absence of any commercial or financial relationships that could be construed as a potential conflict of interest.

## Publisher’s Note

All claims expressed in this article are solely those of the authors and do not necessarily represent those of their affiliated organizations, or those of the publisher, the editors and the reviewers. Any product that may be evaluated in this article, or claim that may be made by its manufacturer, is not guaranteed or endorsed by the publisher.

## Supplementary Material

Supplementary Figure 1 Sample clustering analysis **(A)** and clustering diagram **(B)** of GSE56815; Sample clustering analysis **(C)** and clustering diagram **(D)** of GSE28829. OP: osteoporosis; CON: control; AAS: advanced atherosclerosis; EAS: early atherosclerosis.

Supplementary Figure 2 Volcanic map of DEGs (|logFC| > 1 and adj. p-value < 0.05) in GSE35958 **(A)** and in GSE100927 **(B)**.

Supplementary Figure 3 The functional enrichment analysis of eight common miRNAs through HMDD database.
