# Supplementary figures and images for: Integrated Bioinformatic Analysis of the Shared Molecular Mechanisms Between Osteoporosis and Atherosclerosis
Source: Front Endocrinol (Lausanne). 2022 Jul 22;13:950030. doi: 10.3389/fendo.2022.950030 (PMC9353191; doi:10.3389/fendo.2022.950030)

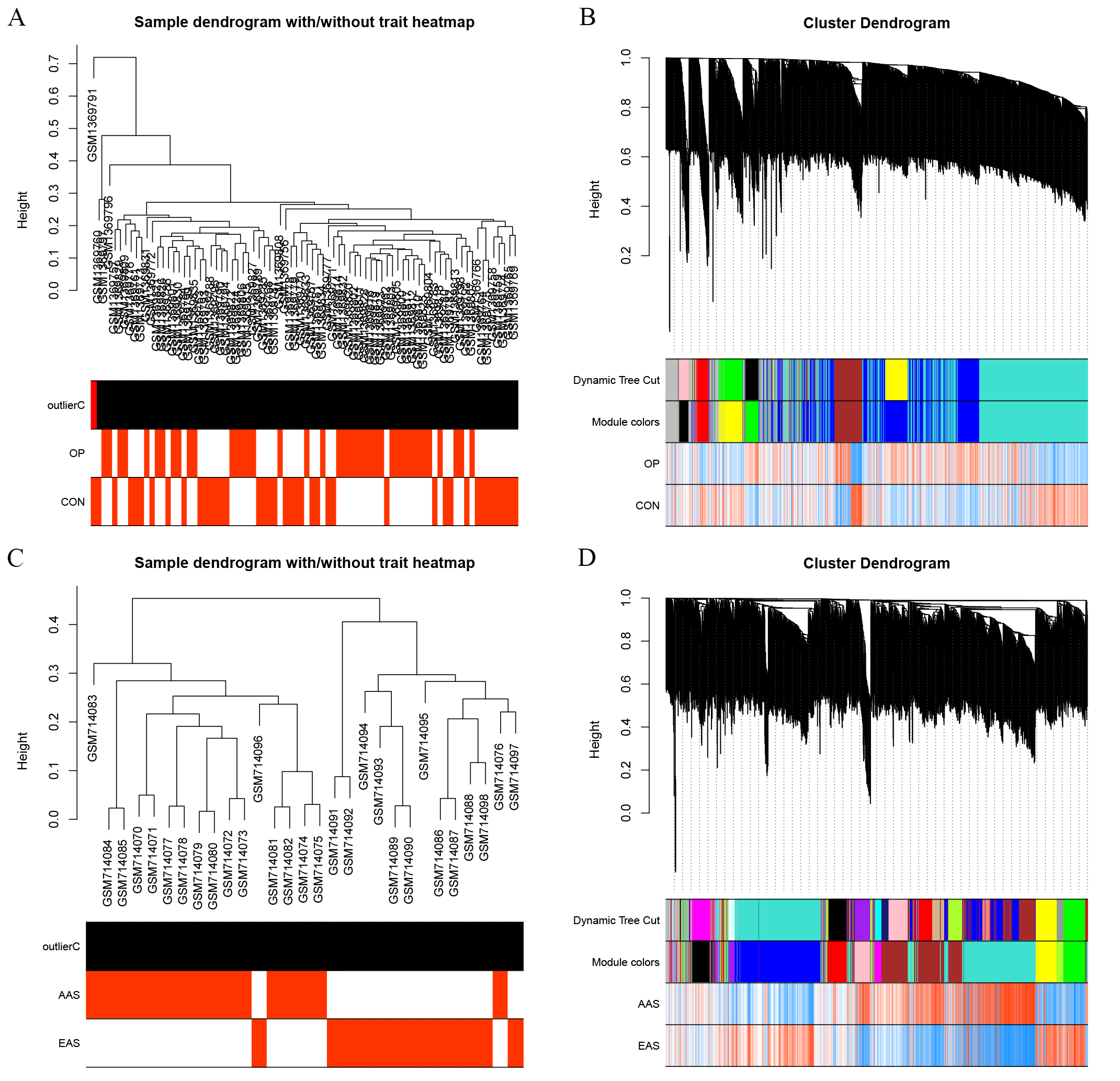

Supplement: Supplementary Figure 1 — Sample clustering analysis (A) and clustering diagram (B) of GSE56815; Sample clustering analysis (C) and clustering diagram (D) of GSE28829. OP: osteoporosis; CON: control; AAS: advanced atherosclerosis; EAS: early atherosclerosis. [file Image_1.tif]

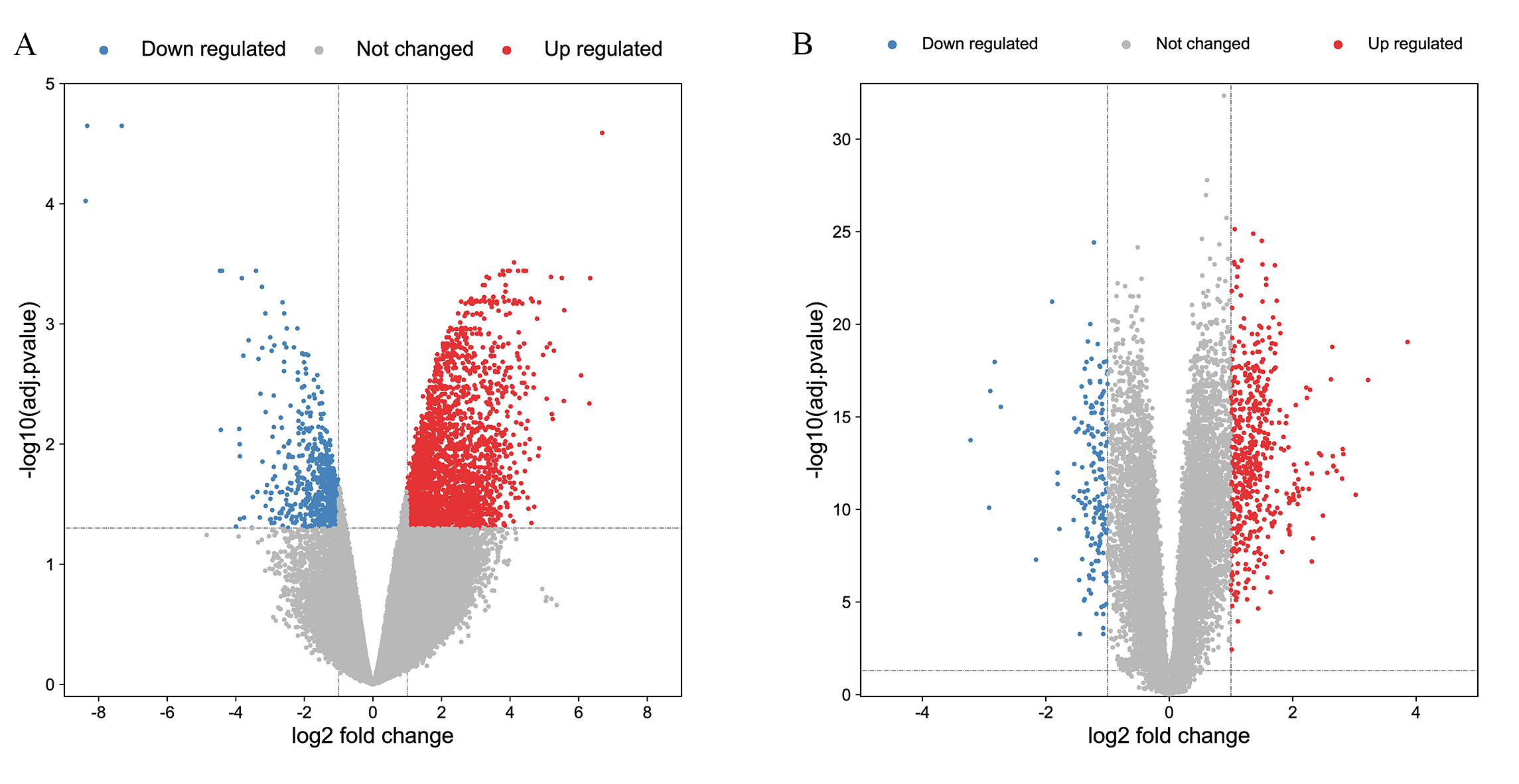

Supplement: Supplementary Figure 2 — Volcanic map of DEGs (|logFC| > 1 and adj. p-value < 0.05) in GSE35958 (A) and in GSE100927 (B). [file Image_2.tif]

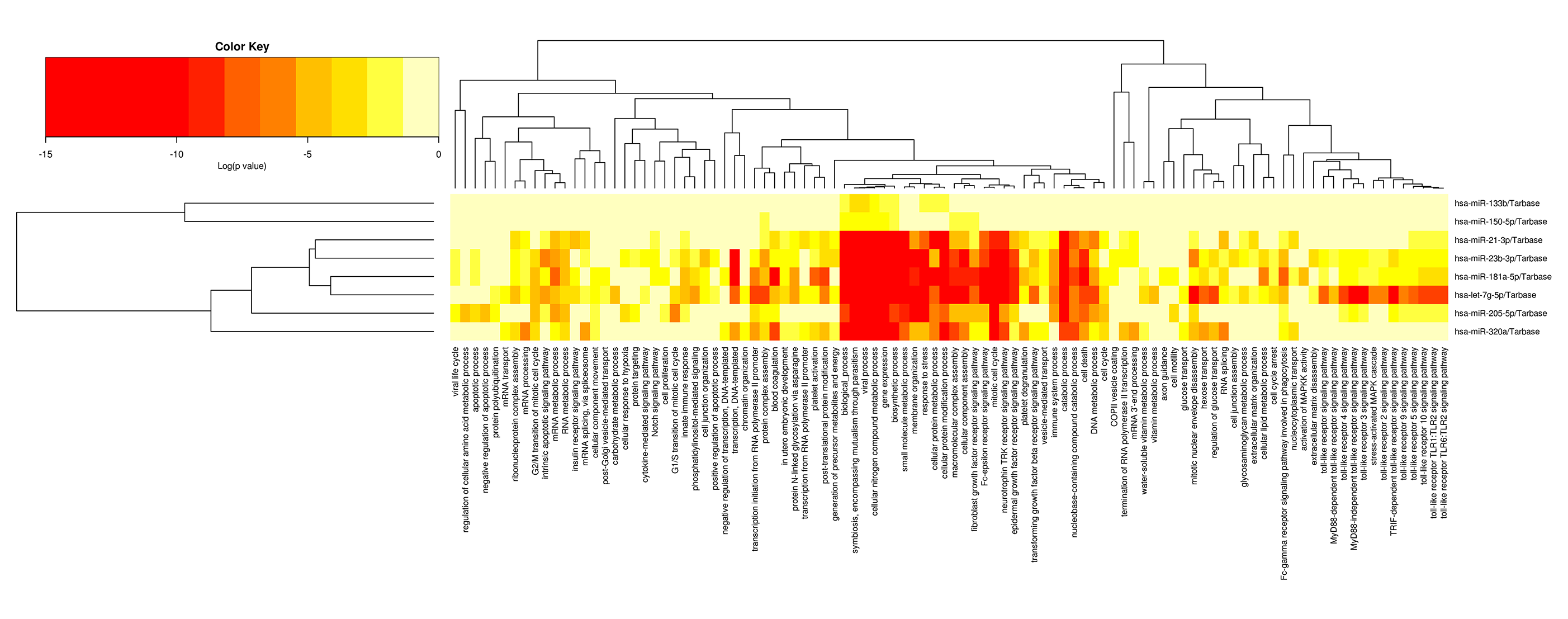

Supplement: Supplementary Figure 3 — The functional enrichment analysis of eight common miRNAs through HMDD database. [file Image_3.tif]
