# Supplementary material for: Integrated Bioinformatic Analysis of the Shared Molecular Mechanisms Between Osteoporosis and Atherosclerosis
Source: Front Endocrinol (Lausanne). 2022 Jul 22;13:950030. doi: 10.3389/fendo.2022.950030 (PMC9353191; doi:10.3389/fendo.2022.950030)
Supplement: Supplementary file 4 [file Table_1.docx]

Supplementary Table1: The list of the common target genes of osteoporosis and atherosclerosis from three public databases.

| APOE | EFNB2 | RORA | GDF15 | KITLG | MMP8 | GRN | PON2 | PLEK |
| --- | --- | --- | --- | --- | --- | --- | --- | --- |
| APOB | RAC2 | CFTR | C3 | SMPD2 | LRP6 | TERF2 | LOX | TPH1 |
| APOA1 | CTSC | CYP27B1 | CYP2C9 | MAPK12 | VCL | MTNR1B | FABP4 | PEPD |
| CRP | INHBA | CD79A | SLC10A2 | RALA | MAPK14 | BMP4 | TGFB3 | LIF |
| CETP | CDK4 | TNFRSF10A | LGALS3 | GDF11 | TGFBR3 | MEFV | TGFB1 | MYOD1 |
| CCL2 | ACVR2A | FST | ALDH2 | KLRK1 | MAPK1 | CD4 | LRP1 | STUB1 |
| PON1 | HDAC7 | AMH | CD68 | FGF4 | HGF | JAG1 | MGP | KDM6B |
| SERPINE1 | TTPA | SLC11A1 | ALOX12 | CTSG | DCN | KCNQ1 | ABCC6 | IAPP |
| APOC3 | CYP24A1 | TCN2 | CSF3 | MYH9 | TNFRSF1A | EPO | NAMPT | OXT |
| CYP27A1 | NR1D1 | CHGA | NFKBIA | NGFR | THBS1 | MDM2 | ERCC1 | APC |
| MPO | CCNB1 | NOTCH2 | VTN | TNFRSF13B | GCLC | OSM | XYLT1 | SIRT2 |
| VWF | ENO1 | ITGAV | GCK | POU5F1 | NFKB1 | CMA1 | TNNT2 | MAPK13 |
| RETN | ENPP2 | TRAF2 | TTR | HDAC5 | FGF23 | COL1A1 | PLAT | SULT2A1 |
| HNF1A | F9 | SLC40A1 | CSK | CEACAM5 | CCND1 | SLC10A1 | PKD2 | MIF |
| COMP | SULT1E1 | SMPD3 | CX3CL1 | DLX5 | MMP2 | IGFBP2 | IGFBP5 | MSTN |
| EPOR | SLC6A4 | TWIST1 | IL1RN | CEACAM1 | NR1H2 | GAPDH | GNA11 | SELL |
| SOX9 | HOXC6 | RARA | TLR2 | FOXA1 | HMOX1 | GGCX | MSX2 | IL2 |
| GOT2 | CTSD | GHSR | IL4 | SKP1 | TIMP1 | APEX1 | PPP3R1 | CHI3L1 |
| PPP1R15A | USF2 | IFNG | SIRT1 | IL5 | HSPB1 | MMP7 | SERPINF1 | RBX1 |
| LYVE1 | GZMB | IBSP | DUSP1 | IRF1 | THPO | DLL4 | NOD1 | KEAP1 |
| TBX21 | AEBP1 | LMNB1 | EIF2S1 | NR3C1 | PTGIS | M6PR | CDKN1B | PNPLA3 |
| MMP13 | ABCG2 | XBP1 | GSR | PDGFRA | TNFSF10 | SPARC | PRTN3 | SORT1 |
| NT5E | CD38 | TG | RGS2 |  |  |  |  |  |

Supplementary Table 2: The top 20 results of KEGG enrichment analysis of 202 common targets.

| No. | Term | Description | LogP | Count |
| --- | --- | --- | --- | --- |
| 1 | hsa05200 | Pathways in cancer | -26.0067 | 37 |
| 2 | hsa04060 | Cytokine-cytokine receptor interaction | -23.2948 | 28 |
| 3 | hsa04151 | PI3K-Akt signaling pathway | -18.816 | 26 |
| 4 | hsa04658 | Th1 and Th2 cell differentiation | -17.7534 | 16 |
| 5 | hsa05142 | Chagas disease | -16.9994 | 16 |
| 6 | hsa04659 | Th17 cell differentiation | -15.145 | 15 |
| 7 | hsa05418 | Fluid shear stress and atherosclerosis | -14.793 | 16 |
| 8 | hsa04933 | AGE-RAGE signaling pathway in diabetic complications | -14.2186 | 14 |
| 9 | hsa05417 | Lipid and atherosclerosis | -14.1197 | 18 |
| 10 | hsa05163 | Human cytomegalovirus infection | -13.775 | 18 |
| 11 | hsa05144 | Malaria | -13.5686 | 11 |
| 12 | hsa04657 | IL-17 signaling pathway | -13.1689 | 13 |
| 13 | hsa04350 | TGF-beta signaling pathway | -13.1689 | 13 |
| 14 | hsa05140 | Leishmaniasis | -12.8406 | 12 |
| 15 | hsa04010 | MAPK signaling pathway | -12.8342 | 19 |
| 16 | hsa04660 | T cell receptor signaling pathway | -12.5855 | 13 |
| 17 | hsa04668 | TNF signaling pathway | -12.1622 | 13 |
| 18 | hsa05205 | Proteoglycans in cancer | -12.1313 | 16 |
| 19 | hsa05170 | Human immunodeficiency virus 1 infection | -11.9069 | 16 |
| 20 | hsa01522 | Endocrine resistance | -11.5493 | 12 |

Supplementary Table 3: The top 20 results of biological processes (BP) enrichment analysis of 202 common targets.

| No. | Term | Description | LogP | Count |
| --- | --- | --- | --- | --- |
| 1 | GO:0009725 | response to hormone | -32.5328 | 48 |
| 2 | GO:0034097 | response to cytokine | -32.162 | 49 |
| 3 | GO:1901652 | response to peptide | -27.928 | 36 |
| 4 | GO:0071345 | cellular response to cytokine stimulus | -26.9464 | 42 |
| 5 | GO:0071396 | cellular response to lipid | -25.8128 | 36 |
| 6 | GO:0001934 | positive regulation of protein phosphorylation | -25.4373 | 41 |
| 7 | GO:0045596 | negative regulation of cell differentiation | -25.2533 | 40 |
| 8 | GO:0050865 | regulation of cell activation | -23.2599 | 38 |
| 9 | GO:0007167 | enzyme linked receptor protein signaling pathway | -23.0489 | 36 |
| 10 | GO:0001819 | positive regulation of cytokine production | -22.0034 | 32 |
| 11 | GO:0001944 | vasculature development | -21.7289 | 33 |
| 12 | GO:0030155 | regulation of cell adhesion | -21.5861 | 38 |
| 13 | GO:0001568 | blood vessel development | -21.2485 | 32 |
| 14 | GO:0032102 | negative regulation of response to external stimulus | -21.1449 | 30 |
| 15 | GO:0008285 | negative regulation of cell population proliferation | -21.1168 | 38 |
| 16 | GO:0048534 | hematopoietic or lymphoid organ development | -21.0418 | 35 |
| 17 | GO:0007568 | aging | -20.6662 | 25 |
| 18 | GO:0070848 | response to growth factor | -20.433 | 31 |
| 19 | GO:0002520 | immune system development | -20.1954 | 35 |
| 20 | GO:0050727 | regulation of inflammatory response | -19.8699 | 28 |
